# The Alzheimer’s Disease Sequencing Project: Progress and Update

**DOI:** 10.1002/alz.093135

**Published:** 2025-01-03

**Authors:** Margaret A Pericak‐Vance

**Affiliations:** ^1^ 1501 NW 10th Avenue, Miami, FL USA

## Abstract

In response to the National Alzheimer’s Project Act (NAPA) in 2012 the National Institute on Aging (NIA) commenced a new genetics initiative (the Alzheimer’s Disease Sequencing Project [ADSP]) to understand the genetic architecture of Alzheimer’s Disease and Related Dementias (AD/ADRD). The ADSP has three main goals 1) To Identify new genes and genetic variations that contribute to increased risk for or protection against AD/ADRD, 2) to provide insight as to how these genes and variations impact AD/ADRD, 3) to identify potential avenues or approaches to translate genetic results into therapeutic targets. The ADSP has led the field in global inclusion and diversity with the goal of universal success in the treatment and prevention of AD/ADRD. The most recent release of over 36,000 genomes from over 40 cohorts worldwide is evidence to this effect. This first presentation will be a brief introduction and overview of the structure, growth, and progress of the ADSP.

